# Combined Snail and E-cadherin Predicts Overall Survival of Cervical Carcinoma Patients: Comparison Among Various Epithelial-Mesenchymal Transition Proteins

**DOI:** 10.3389/fmolb.2020.00022

**Published:** 2020-02-28

**Authors:** Yuejun Tian, Ping Qi, Qian Niu, Xuemei Hu

**Affiliations:** ^1^Department of Obstetrics and Gynecology, Lanzhou University Second Hospital, Lanzhou, China; ^2^Department of Clinical Laboratory, Lanzhou University Second Hospital, Lanzhou, China; ^3^Department of Pathology, Lanzhou University Second Hospital, Lanzhou, China

**Keywords:** EMT, Snail, E-cadherin, prognosis, cervical carcinoma

## Abstract

**Background:**

Activation of Snail and synergistic loss of E-cadherin are hallmark features of the epithelial-mesenchymal transition (EMT), which contributes to the metastasis phenotype of epithelial cancer cells. However, the prognostic impact of Snail and of its combination with E-cadherin and with other EMT prognostic markers has not yet been systematically studied in cervical carcinoma. This study aimed to explore the prognostic value of combined Snail and E-cadherin in patients with cervical carcinoma and compared it to the prognostic value of other EMT prognostic markers.

**Methods:**

We retrospectively identified every initial diagnosis of cervical carcinoma among 203 patients treated at our hospital in China from January 2008 to March 2013. We examined the prognostic significance of Snail and other EMT protein markers, such as E-cadherin, Slug, ZEB1, Twist, Vimentin, and Survivin, by univariate and multivariate survival analyses.

**Results:**

Multivariate analyses showed that Snail and E-cadherin were significant biomarkers for overall survival (OS) in cervical carcinoma patients (HR, hazard ratio = 1.744, *P* = 0.036 and HR = 1.738, *P* = 0.047; respectively). Moreover, a combined index including Snail and E-cadherin showed enhanced prognostic value compared to that of Snail or E-cadherin alone. The present data demonstrate that Snail shows a negative correlation with E-cadherin (*P* < 0.001). High Snail expression and low E-cadherin expression were also more common in high tumor stages (*P* = 0.044 and *P* = 0.036; respectively), and lymph node metastasis (both *P* < 0.001). Moreover, Snail was a superior prognosis factor compared to Slug, ZEB1, Twist, Vimentin, and Survivin in cervical carcinoma.

**Conclusion:**

Based on our results, Snail and E-cadherin may be considered as independent prognosis markers, and the combination of Snail and E-cadherin might improve the OS prediction accuracy for patients with cervical carcinoma.

## Introduction

As it is one of the four most common malignancies in women, cervical carcinoma threatens the health of women around the world (Bray et al., 2018). Despite the development of effective vaccines for prevention, patients with metastatic disease and advanced disease still have a poor prognosis, with a 5-year survival rate for metastatic cervical carcinoma of only 16.5% (Ferlay et al., 2013; Li H. et al., 2016). Thus, novel prognostic biomarkers for cervical carcinoma are urgently needed.

Epithelial-mesenchymal transformation (EMT) is characterized by increased cell mobility and loss of cell adhesion, which are closely related to tumor invasion and metastasis (Brabletz et al., 2018; Pastushenko and Blanpain, 2019). EMT is a complicated and multi-step process that has been found to be initiated, regulated, and maintained by several factors, including hypoxia, cytokines, signaling pathways, and transcription factors in cervical carcinoma.

Intra-tumoural hypoxia is commonly found in carcinoma, and hypoxia-induced proteome changes promote cervical carcinoma cell invasion and migration by participating in tumor necrosis factor (TGF)-β1-induced EMT (Wilson and Hay, 2011). Hypoxia can enhance ZEB1 expression and promote cervical carcinoma progression through increased CCL8 secretion and tumor-associated macrophage recruitment (Chen et al., 2019). Other EMT inducers include the inflammatory-based cytokines [i.e., interleukin (IL)-6, TNF-α, TGF-β]. IL-6, one of the key cytokines in the tumor microenvironment, and its stimulation induce the EMT program in cervical carcinoma cells (Miao et al., 2014). Given its established role in tumor carcinogenesis and metastasis, TNF-α is one of the essential EMT-promoting cytokines in a variety of tumors, including cervical carcinoma (Bohrnsen et al., 2019; Dong et al., 2019; Tan et al., 2019; Cruceriu et al., 2020). TGF-β, which is produced and secreted by tumor-infiltrating immune cells, can induce EMT in an advanced cervical tumor model by 3D printing (Sistigu et al., 2017). Thus, reactivation of EMT also links inflammation-based cytokines to cervical carcinoma. Moreover, EMT is involved in all types of signaling pathways (e.g., NF-kB, EGFR–ERK, BMP, Wnt/β-catenin, Akt/GSK-3b/Snail, EGF/EGFR, p53/TIGAR and SCO2 pathways), which are regulated by the zinc-finger family of transcription factors, including Snail, Slug, and Twist, which contribute to tumor metastasis by promoting EMT in cervical carcinoma (Qureshi et al., 2015).

E-cadherin (CDH1) is one of the most important components of adherens junctions, which are integral in cell adhesion and are principal organizers of the epithelial phenotype (van Roy, 2014). EMT contributes to enhanced mobility and the invasion of epithelial cells that results in the evolution of tumors toward migration and metastasis (Thiery, 2002). It is well established that loss of E-cadherin is involved in EMT, which induces the migration and metastasis of tumor cells, including cervical carcinoma (Schmalhofer et al., 2009; Liu et al., 2017; Jing et al., 2019). Snail, a main EMT-activating transcriptional factor, controls EMT by repressing E-cadherin expression, and upregulation of its expression is associated with a poor prognosis in many types of carcinoma (Zhang et al., 2014; Fazilaty et al., 2019). Snail was significantly upregulated in cervical squamous cell carcinoma, and high levels can contribute to the onset of EMT; however, its effectiveness in prognosis prediction of cervical carcinoma has not been fully characterized (Zhao et al., 2013; Gong et al., 2015).

Transcription factors, including Snail family members (Slug), Twist, ZEB1 and others, have been identified as critical regulators of EMT during tumor progression, embryogenesis, and metastasis (Krebs et al., 2017; Wu et al., 2019). Slug and Twist, representing basic helix–loop–helix transcription factors, act as cell–cell adhesion disrupters by inhibiting E-cadherin expression (El Ghouzzi et al., 2000; De Craene et al., 2005; Qin et al., 2012). ZEB1 has two zinc-finger domains, located at the N and C termini, through which it binds to E-box-like sequences (CACCTG) in target DNA (Ikeda and Kawakami, 1995). ZEB1 functions act as a transcriptional repressor of cell–cell adhesion by suppressing the glycoprotein E-cadherin.

EMT involves the loss of E-cadherin expression and acquisition of the expression of the mesenchymal marker Vimentin (Tiwari et al., 2012). Vimentin, which serves as an important intermediate filament protein in mesenchymal cells, plays a key role in the metastatic growth and invasion of tumor cells, including cervical carcinoma cells (Cheng et al., 2012; Lu et al., 2019). Survivin, a member of the inhibitors of apoptotic protein (IAP) family, which suppress apoptosis and enhance tumor cell proliferation and angiogenesis, is implicated in the EMT of some tumors (Mita et al., 2008; Breyer et al., 2016; Liu et al., 2018; Zhao et al., 2019).

The purpose of this pilot study was to clarify whether expression of Snail, as determined by immunohistochemistry, can help predict the long-term survival outcome of cervical carcinoma patients and to compare its value to that of an epithelial marker (E-cadherin), transcription factors (Slug, ZEB1, Twist), a mesenchymal marker (Vimentin), and another marker (Survivin). We found that although low expression of E-cadherin and high expression of Snail, Slug, Twist, Vimentin, and Survivin indicated a worse prognosis, only E-cadherin and Snail independently predicted survival after adjusting for confounding variables.

## Materials and Methods

### Patients

We analyzed 203 cervical carcinoma samples collected from operable cervical cases and 56 pair-matched adjacent normal tissues (peritumoral tissue more than 2 cm from cancer tissue). All clinical cervical carcinoma samples were collected from the Department of Pathology at the Second Hospital of Lanzhou University between January 2008 to March 2013. The samples were used in accordance with the principles of the Declaration of Helsinki and the guidelines of the Second Hospital of Lanzhou University Ethical Review Board for Medical Research Involving Human Subjects (approval number: 2016A-072). Written informed consent was obtained from all patients.

Patients’ clinical parameters and baseline data were collected, including International Federation of Gynecology and Obstetrics (FIGO) stage, age, lymph node metastasis status, tumor type, and histologic grade. All patients had access to their clinical follow-up data, and none had received biotherapy, chemotherapy or radiotherapy prior to surgery. The clinical stage of cervical carcinoma patients was determined according to the FIGO staging system established in 2009. The patients’ median age was 55 years (range = 28–82 years). The basic characteristics of the population are summarized in Table 1. Patients with cervical carcinoma were generally followed up via telephone or postoperative visits. Overall survival (OS) refers to the period from the date of diagnosis to the date of the last follow-up visit (June 2018) or the date of death.

**TABLE 1:** Correlation of Snail and E-cadherin expression with the clinicopathologic characteristics of 203 cervical carcinoma patients.

**Variable**	**Snail**			**E-cadherin**		
	**High**	**Low**	***p*-value**	**High**	**Low**	***p*-value**
**Age (years)**						
<55	55	34	0.843	32	57	0.374
≥55	72	42		48	66	
**FIGO stage**						
I–II	60	47	0.044	51	56	0.011
III–IV	67	29		29	67	
**Histological grade**						
Well/moderately differentiated (G1-G2)	77	51	0.355	55	73	0.175
Poorly differentiated (G3)	50	25		25	50	
**Lymph node metastasis**						
No	60	58	<0.001	68	50	<0.001
Yes	67	18		12	73	
**Histological type**						
SCC	105	59	0.377	64	100	0.818
other (AD/ASC)	22	17		16	23	

*AD, adenocarcinoma; ASC, adenosquamous cell carcinoma; FIGO, International Federation of Gynecology and Obstetrics; SCC, squamous cell carcinoma. Bold font indicates *p*-values < 0.05.*

### Immunohistochemical Staining

Immunohistochemistry was carried out following the manufacturer’s recommendations. Tissue sections were embedded in paraffin and deparaffinized with xylene for 15 min, fixed with 100% ethanol for 10 min, and then rehydrated. The prepared slices were treated with methanol solution containing 3% hydrogen peroxide for 10 min to block endogenous peroxidase activity. Sections were washed twice with PBS and then incubated with antibodies against Snail, E-cadherin, Slug, ZEB1, Twist, Vimentin and Survivin overnight (dilutions 1:200, 1:100, 1:100, 1:50, 1:75, 1:200, and 1:100) at 4°C. All sections were subjected to a heat-induced antigen retrieval process. Sections were again washed with PBS twice and then incubated with the corresponding secondary antibody under ambient conditions for 30 min. Then, after color development with 3-amino9-ethylcarbazole for 15 min, the slides were counterstained with hematoxylin. Finally, an optical microscope was used to observe the slides. Negative and positive controls were used to optimize staining. EMT inducers (Snail, Slug, ZEB1, and Twist) and EMT markers (E-cadherin, Vimentin, and Survivin) were also used. Rabbit anti-human polyclonal antibodies against Snail (ab180714), E-cadherin (ab15148), Slug (ab180714), ZEB1 (ab87280), Twist (ab49254), Vimentin (ab45939), and Survivin (ab469) were purchased from Abcam (Cambridge, United Kingdom).

### Evaluation of the Immunohistochemistry Results

The immunohistochemical staining scoring system was evaluated according to previously described criteria, with minor modifications (Li et al., 2012; Song et al., 2012). The scores from the proportion of positively stained immunoreactive cells and the staining intensity were added to the overall score.

The staining results of Snail proteins, Slug, ZEB1, Twist, Vimentin, and Survivin in the nucleus and cytoplasm were subsequently evaluated. The amounts of E-cadherin distributed in the membrane and cytoplasm were determined. The intensity scores were determined on a scale from 0 to 3, indicating negative, weak, mild and strong staining, respectively. The proportions of scores were 0 (negative); 1 (1–10% positive cells), 2 (10–70% positive cells), and 3 (>70% positive cells). The total scores ranged from 0 to 6 and were designated 0–2 (low) or 3–6 (high).

### Statistical Analysis

The software used for statistical analysis was SPSS 22.0. The chi-square test was applied to analyze the correlations between E-cadherin and Snail protein expression levels and clinicopathological features. The associations between the expression levels of Snail, E-cadherin, Slug, ZEB1, Twist, Vimentin and Survivin and cervical carcinoma prognosis were analyzed using Kaplan-Meier survival analysis, and the logarithmic rank test was used in the univariate analysis. The significance levels of variables that were independently associated with OS were determined using the Cox proportional hazards regression model in the univariate and multivariate analyses. *P-*values lower than 0.05 were considered statistically significant.

## Results

### Correlation of EMT Protein Expression in Human Cervical Carcinoma Tissue

**FIGURE 1:**
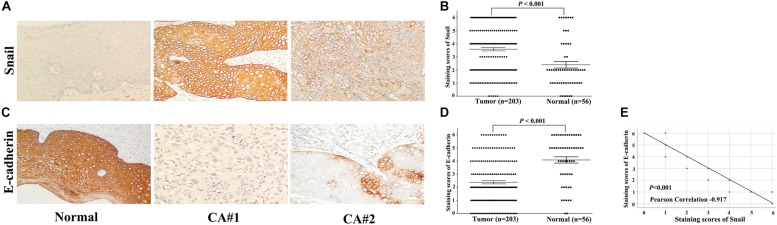
Snail and E-cadherin expression in cervical carcinoma tissues determined by immunohistochemical staining (original magnification × 200). **(A)** Representative Snail expression in tumor and normal tissues, with positive expression located in the cytoplasm. **(B)** Scatter dot plot showing the staining score (mean ± SEM) of Snail in tumor and normal tissues using the paired *t*-test. *P < 0.001; **(C)** Representative E-cadherin expression in tumor and normal tissues, with positive expression located in the membrane and cytoplasm. **(D)** Scatter dot plot showing the staining score (mean ± SEM) of E-cadherin in tumor and normal tissues using the paired *t*-test. *P < 0.001; **(E)** Snail expression was negatively correlated with E-cadherin expression in 203 patients with cervical carcinoma.

We determined Snail and E-cadherin protein expression levels in 203 cases of cervical carcinoma and 56 cases of adjacent normal tissues by IHC staining. The representative IHC results are shown in Figure 1. In total, scatter dot plot showed that the average immunostaining score of Snail protein in 203 tumor tissues was 3.59 ± 1.86; that in 56 normal tissues was 2.39 ± 1.87 (Figures 1A,B, *P* < 0.001). Snail showed a significant difference (high/low expression 127/203 vs. 17/56, *P* < 0.001) (Supplementary Table S1). In contrast, scatter dot plot showed that the average immunostaining score of E-cadherin protein in 203 tumor tissues was 2.38 ± 1.76, whereas that in 56 normal tissues was 4.09 ± 1.78 (Figures 1C,D, *P* < 0.001). E-cadherin protein expression was downregulated in cervical carcinoma compared with normal tissues (low/high expression 80/203 vs. 36/56, *P* = 0.001) (Supplementary Table S1). Moreover, Snail protein upregulation was consistent with E-cadherin downregulation, as shown in serial sections (Figure 1E, *P* < 0.001).

IHC staining also showed that other transcription factors and markers, including Slug, ZEB1, Twist, Vimentin, and Survivin proteins, were more highly expressed in cervical carcinoma compared with normal tissues, at 55.2% (112/203) vs. 48.2% (27/56), *P* = 0.355; 51.2% (104/203) vs. 42.9% (24/56), *P* = 0.267; 53.2% (108/203) vs. 41.1% (23/56), *P* = 0.108; 55.7% (113/203) vs. 32.1% (18/56), *P* = 0.002; and 56.7% (115/203) vs. 42.9% (24/56), *P* = 0.067, respectively (Supplementary Table S1). Scatter dot plot showed that the staining score (mean ± SD) of other transcription factors and markers, including Slug, ZEB1, Twist, Vimentin, and Survivin, were increased in cervical carcinoma compared with normal tissues (high/low score 3.21 ± 1.80 vs. 2.93 ± 1.74, *P* = 0.29; high/low score 3.14 ± 1.72 vs. 2.66 ± 1.83, *P* = 0.08; high/low score 3.22 ± 1.68 vs. 2.34 ± 1.94, *P* = 0.002; high/low score 3.12 ± 1.73 vs. 2.63 ± 1.95, *P* = 0.09, respectively) (Supplementary Figure S1).

### Association of EMT Protein Expression With the Clinicopathological Characteristics of Cervical Carcinoma Patients

Among the 203 patients, Snail overexpression and downregulation of E-cadherin expression showed statistically significant correlations with an aggressive FIGO stage (*P* = 0.044 and *P* = 0.036; respectively), and lymph node metastasis (both *P* < 0.001) (Table 1). Enhanced levels of Slug and Survivin also showed statistically significant correlations with an aggressive FIGO stage (both *P* < 0.001) and lymph node metastasis (both *P* < 0.001). Furthermore, Twist and Vimentin were significantly elevated in patients who had cervical carcinoma with an aggressive FIGO stage (both *P* < 0.001), a high histological grade (*P* = 0.003 and *P* = 0.016; respectively), and lymph node metastasis (both *P* < 0.001) (Supplementary Table S2).

### Univariate and Multivariate Survival Analyses of all Prognostic Parameters

We evaluated the effect of Snail, Slug, ZEB1, Twist, Vimentin, Survivin, and E-cadherin expression on OS using a Cox proportional hazard regression model (Table 2). The risk of death was assessed using univariate analysis. The results indicated that FIGO stage (HR = 2.490, *P* < 0.001), histological grade (HR = 1.575, *P* = 0.041), lymph node metastasis (HR = 3.341, *P* < 0.001), Snail (HR = 2.238, *P* = 0.001), Slug (HR = 1.785, *P* = 0.012), Twist (HR = 2.131, *P* = 0.001), Vimentin (HR = 2.226, *P* = 0.001), Survivin (HR = 1.791, *P* = 0.012), and E-cadherin (HR = 2.612, *P* < 0.001) were significantly associated with an increased risk of death. After adjusting for confounding variables, multivariate analysis showed that FIGO stage (HR = 1.782, *P* = 0.029), lymph node metastasis (HR = 1.887, *P* = 0.025), Snail (HR = 1.744, *P* = 0.036), and E-cadherin (HR = 1.738, *P* = 0.047) correlated obviously with OS (Table 2).

**TABLE 2:** Univariate and multivariate analyses of the characteristics associated with OS in 203 cervical carcinoma patients.

**Characteristics**	**Univariate**			**Multivariate**		
	**HR**	**95% CI**	***p*-value**	**HR**	**95% CI**	***p*-value**
**Age, years**	0.818	0.530–1.263	0.365	0.741	0.478–1.150	0.181
**Histologic type**						
AD/ASC	1.00 (ref.)			1.00 (ref.)		
SCC	1.256	0.706–2.234	0.438	1.301	0.709–2.387	0.395
**FIGO stage**						
I–II	1.00 (ref.)			1.00 (ref.)		
III–IV	2.490	1.588–3.905	<0.001	1.782	1.060–2.995	0.029
**Histologic grade**						
G1-G2	1.00 (ref.)			1.00 (ref.)		
G3	1.575	1.018–2.436	0.041	1.313	0.835–2.064	0.239
**Lymph node metastasis**						
No	1.00 (ref.)			1.00 (ref.)		
Yes	3.341	2.115–5.276	<0.001	1.887	1.083–3.287	0.025
**Snail expression**						
Low	1.00 (ref.)			1.00 (ref.)		
High	2.238	1.366–3.668	0.001	1.744	1.038–2.928	0.036
**Slug expression**						
Low	1.00 (ref.)			1.00 (ref.)		
High	1.785	1.133–2.813	0.012	0.727	0.410–1.288	0.274
**ZEB1 expression**						
Low	1.00 (ref.)			1.00 (ref.)		
High	1.181	0.765–1.822	0.128	1.239	0.749–2.051	0.404
**Twist expression**						
Low	1.00 (ref.)			1.00 (ref.)		
High	2.131	1.341–3.386	0.001	0.926	0.434–1.980	0.844
**Vimentin expression**						
Low	1.00 (ref.)			1.00 (ref.)		
High	2.226	1.387–3.573	0.001	1.467	0.828–2.599	0.189
**Survivin expression**						
Low	1.00 (ref.)			1.00 (ref.)		
High	1.791	1.135–2.827	0.012	1.348	0.737–2.465	0.332
**E-cadherin expression**						
Low	1.00 (ref.)			1.00 (ref.)		
High	2.612	1.583–4.309	<0.001	1.738	1.008–2.998	0.047

*AD, adenocarcinoma; ASC, adenosquamous cell carcinoma; CI, confidence interval; FIGO, International Federation of Gynecology and Obstetrics; HR, hazard ratio; SCC, squamous cell carcinoma. Bold font indicates *p*-values < 0.05.*

**FIGURE 2:**
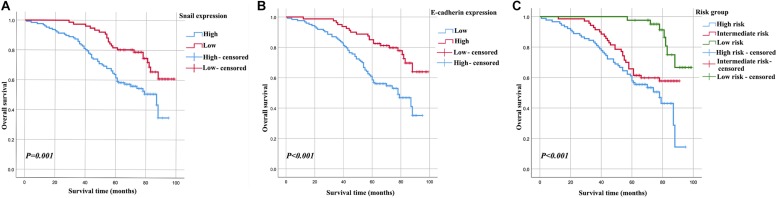
Kaplan–Meier curves for 5-year OS rate of patients with cervical carcinoma. OS based on Snail in cervical carcinoma patients **(A)**, OS based on E-cadherin in cervical carcinoma patients **(B)**, OS of combined Snail and E-cadherin-based categorisation in cervical carcinoma patients (high risk, Snail-high and E-cadherin-low; intermediate risk, Snail-high and E-cadherin-high or Snail-low and E-cadherin-high; and low risk, Snail-low and E-cadherin-high) **(C)**.

In the Kaplan-Meier analyses, increased Snail expression (Figure 2A) and decreased E-cadherin expression (Figure 2B) were unfavorable outcomes for cervical carcinoma patients (all *P* < 0.05). These results were supported by the estimated cumulative 5-year survival rates of cervical carcinoma patients with expression levels of Snail (62.2% high vs. 82.9% low) and E-cadherin (85.0% high vs. 60.9% low).

### Prognostic Value of the Combined Snail and E-cadherin

The relationship between Snail and E-cadherin was performed using Spearman’s rank correlation analysis. Methods combining Snail with E-cadherin might improve patient stratification be related to OS. Thus, the patients were divided into three groups: high risk, Snail-high and E-cadherin-low; intermediate risk, Snail-high and E-cadherin-high or Snail-low and E-cadherin-high; and low risk, Snail-low and E-cadherin-high (Figure 2C). The high-, intermediate- and low-risk groups showed 5-year OS rates corresponding to 60.0, 67.1, and 95.3%, respectively. After adjusting for other clinical variables in multivariate analysis (Table 3), both the high- and intermediate-risk groups had significantly worse prognoses compared with the low-risk group.

**TABLE 3:** Multivariate analyses of the characteristics associated with OS in 203 cervical carcinoma patients.

**OS Multivariate analysis**			
**Characteristics**	**HR**	**95% CI**	***p*-value**
**Age, years**	0.715	0.459–1.114	0.138
**Histologic type**			
SCC	1.00 (ref.)		
AD/ASC	1.387	0.749–2.566	0.289
**FIGO stage**			
I–II	1.00 (ref.)		
III–IV	1.827	1.362–3.090	0.024
**Histologic grade**			
G1-G2	1.00 (ref.)		
G3	1.393	0.890–2.181	0.147
**Lymph node metastasis**			
No	1.00 (ref.)		
Yes	1.804	1.038–3.134	0.036
**Risk group**			
Low risk	1.00 (ref.)		
Intermediate risk	2.767	1.177–6.504	0.020
High risk	3.894	1.647–9.206	0.002

*AD, adenocarcinoma; ASC, adenosquamous cell carcinoma; CI, confidence interval; FIGO, International Federation of Gynecology and Obstetrics; HR, hazard ratio; SCC, squamous cell carcinoma. Bold font indicates *p*-values < 0.05.*

## Discussion

Increasing evidence indicates that up-regulation and nuclear accumulation of Snail correlate with EMT in cervical carcinoma (Lee et al., 2008; Zhao et al., 2013). An et al. (2016) reported that in cervical carcinoma cells, α-Actinin-4 promotes EMT and tumorigenesis by regulating Snail expression and the Akt pathway. Pang et al. (2017) found that YB-1 promotes EMT and the progression of cervical carcinoma by upregulating Snail expression, which suggested that the YB-1/Snail/EMT axis may be used as a potential candidate for the diagnosis and therapy of cervical carcinoma metastasis. However, little is known about the prognosis outcome of Snail in cervical carcinoma, and the values of Snail expression in cervical carcinoma and its clinical significance have not been thoroughly explored.

In our study, the data indicated the following: (1) high Snail expression predicts a lower survival rate of cervical carcinoma patients; (2) high Snail expression is associated with a highly aggressive FIGO stage and LNM status in cervical carcinoma patients; (3) Snail expression is increased in cervical carcinoma tissue compared to normal tissue, and it correlates negatively with E-cadherin expression; (4) the Snail protein is a better prognostic factor than Slug, Twist, ZEB1, Vimentin, and Survivin in cervical carcinoma; and (5) combining Snail and E-cadherin proteins may improve the precision of OS prediction in cervical carcinoma patients.

Low E-cadherin expression is correlated with FIGO stage and lymph node metastasis, and it is a useful marked for survival outcomes in cervical carcinoma. E-cadherin immunostaining might assist in the diagnosis of cervical intraepithelial neoplasia and indicate prognosis in early stage cervical squamous cell carcinoma patients (Li B. et al., 2016). Based on this study, we suggest that E-cadherin may be considered an anti-oncogene and that downregulation of E-cadherin may contribute to tumor metastasis and poor prognosis.

The other transcription factors Slug, Twist, ZEB1 have been confirmed to serve as useful prognostic factors in many cancers. The levels of Slug, Twist, and ZEB1 expression between tumor tissues and normal tissues were not significant (Supplementary Figure S1), and multivariate analysis demonstrated that Slug, Twist, ZEB1 were not independent prognostic factors in cervical carcinoma. However, Wushou et al. (2014) and Huang et al. (2016) showed that expression of Twist and Slug is associated with worse survival in carcinoma. Our univariate analysis results showed high Twist and Slug expression levels are closely related to shorter OS, but no significant relationship was found between OS and ZEB1 expression. Therefore Snail might be a more effective EMT-related marker than Slug, Twist, and ZEB1 for predicting survival.

Increased Vimentin expression corresponded with FIGO stage, the grade of differentiation and LNM. Overexpression Vimentin clearly predicted shorter OS in univariate analysis but not in multivariate analysis. This is different from the findings of other studies. Lin et al. (2017) showed in a multivariate analysis that Vimentin overexpression in cervical cancer patients was significantly associated with poor OS. The differences may be due to differences in sample size by analysis by the log-rank test in the previous study. High Survivin expression was a prognostic factor for a short OS in univariate Cox regression analysis but not in multivariate analysis. In addition, the level of Survivin expression did not achieve a meaningful difference between tumor tissue and normal tissue. The result suggests that Survivin is a prognostic factor of OS of patients with cervical carcinoma but not an independent prognostic factor.

The present data showed a relationship between Snail and E-cadherin. Snail is a zinc finger transcription factor that mainly participates in EMT by upregulating expression of E-cadherin in epithelial tumor cells (Lin et al., 2014; Liu et al., 2019). This finding of a strong correlation between Snail and E-cadherin further suggests complex interaction between the transcription factors, with EMT biomarkers potentially influencing patient survival.

The combination of high Snail and low E-cadherin levels resulted in the lowest 5-year survival rate among the three groups. These results further support that Snail and E-cadherin are related to EMT in cervical carcinoma, leading to a subsequent adverse outcome, and further confirm that Snail correlates negatively with E-cadherin. Furthermore, our findings highlight that combining Snail and E-cadherin can discriminate patients with better prognosis in cervical carcinoma compared with Snail or E-cadherin alone.

Our study has some limitations. As this was a retrospective, single-center study, additional studies with larger patient groups are needed to evaluate the potential of these markers.

## Conclusion

We demonstrate that Snail and E-cadherin act as independent factors for predicting OS in cervical carcinoma. Based on the findings of this study, combining Snail and E-cadherin expression can improve the prognostic accuracy and serve as a select criterion for risk factor-stratified patient management in cervical carcinoma.

## Data Availability Statement

The datasets analyzed in this article are not publicly available. Requests to access the datasets should be directed to YT, tianyj14@lzu.edu.cn.

## Ethics Statement

The studies involving human participants were reviewed and approved by the Lanzhou University Second Hospital Ethics Committee. The patients/participants provided their written informed consent to participate in this study. Written informed consent was not obtained from the individual(s) for the publication of any potentially identifiable images or data included in this article.

## Author Contributions

XH conceived the idea. YT, PQ, and QN performed all the experiments and wrote the manuscript.

## Conflict of Interest

The authors declare that the research was conducted in the absence of any commercial or financial relationships that could be construed as a potential conflict of interest.
