# Supplementary material for: Combined Snail and E-cadherin Predicts Overall Survival of Cervical Carcinoma Patients: Comparison Among Various Epithelial-Mesenchymal Transition Proteins
Source: Front Mol Biosci. 2020 Feb 28;7:22. doi: 10.3389/fmolb.2020.00022 (PMC7058927; doi:10.3389/fmolb.2020.00022)
Supplement: Supplementary file 2 [file Table_1.pdf]

SUPPLEMENTARY MATERIAL

TABLE S1 | Expression of Snail, E-cadherin, Slug, ZEB1, Twist, Vimentin, and Survivin in cervical carcinoma and para-carcinoma tissue samples.

Variables	High expression (n)	Low expression (n)	X^2	*P*-value
**Snail**			18.440	< 0.001
Carcinoma (203)	127	76		
Para-carcinoma (56)	17	39		
**E-cadherin**			10.985	0.001
Carcinoma (203)	80	123		
Para-carcinoma (56)	36	20		
**Slug**			0.855	0.355
Carcinoma (203)	112	91		
Para-carcinoma (56)	27	29		
**ZEB1 expression**			1.231	0.267
Carcinoma (203)	104	99		
Para-carcinoma (56)	24	32		
**Twist expression**			2.584	0.108
Carcinoma (203)	108	95		
Para-carcinoma (56)	23	33		
**Vimentin expression**			9.715	0.002
Carcinoma (203)	113	90		
Para-carcinoma (56)	18	38		
**Survivin expression**			3.358	0.067
Carcinoma (203)	115	88		
Para-carcinoma (56)	24	32		
