# Supplementary material for: Combined Snail and E-cadherin Predicts Overall Survival of Cervical Carcinoma Patients: Comparison Among Various Epithelial-Mesenchymal Transition Proteins
Source: Front Mol Biosci. 2020 Feb 28;7:22. doi: 10.3389/fmolb.2020.00022 (PMC7058927; doi:10.3389/fmolb.2020.00022)
Supplement: Supplementary file 3 [file Table_2.pdf]

SUPPLEMENTARY MATERIAL

TABLE S2 | Correlation of Slug, ZEB1, Twist, Vimentin, and Survivin expression with the clinicopathologic characteristics of 203 cervical carcinoma patients.

	n	High expression of Slug n (%)	*P*-value	High expression of ZEB1 n (%)	*P*-value	High expression of Twist n (%)	*P*-value	High expression of Vimentin n (%)	*P*-value	High expression of Survivin n (%)	*P*-value
**Age (years)**											
<55	89	48 (53.9)	0.754	39 (43.8)	0.062	49 (55.0)	0.640	43 (48.3)	0.063	44 (49.4)	0.067
≥55	114	64 (56.1)		65 (57.0)		59 (51.8)		70 (61.4)		71 (62.3)	
**FIGO stage**											
I–II	107	69 (64.5)	<0.001	52 (48.6)	0.428	43 (40.2)	<0.001	43 (40.2)	<0.001	48 (44.9)	<0.001
III–IV	96	43 (44.8)		52 (54.2)		65 (67.7)		70 (72.9)		67 (69.8)	
**Histological grade**											
(G1-G2)	130	67 (52.3)	0.290	60 (46.2)	0.105	58 (44.6)	0.003	63 (48.5)	0.016	70 (53.8)	0.461
(G3)	70	45 (62.9)		44 (62.9)		50 (71.4)		50 (71.4)		45 (64.3)	
**Lymph node metastasis**											
No	118	41 (34.7)	<0.001	55 (46.7)	0.121	46 (39.0)	<0.001	49 (41.5)	<0.001	48 (40.7)	<0.001
Yes	85	71 (83.5)		49 ((57.6)		62 (72.9)		64 (75.3)		67 (78.8)	
**Histological type**											
SCC	164	91 (55.5)	0.853	88 (53.7)	0.156	90 (54.9)	0.326	92 (56.1)	0.799	88 (53.7)	0.078
other (AD/ASC)	39	21 (53.8)		16 (41.0)		18 (46.2)		21 (53.8)		27 (69.2)	

Abbreviations: AD, adenocarcinoma; ASC, adenosquamous cell carcinoma; CI, confidence interval; FIGO, International Federation of Gynecology and Obstetrics; SCC, squamous cell carcinoma. Bold font indicates *P*-values <0.05
