# Supplementary material for: Combined Snail and E-cadherin Predicts Overall Survival of Cervical Carcinoma Patients: Comparison Among Various Epithelial-Mesenchymal Transition Proteins
Source: Front Mol Biosci. 2020 Feb 28;7:22. doi: 10.3389/fmolb.2020.00022 (PMC7058927; doi:10.3389/fmolb.2020.00022)
Supplement: Supplementary file 1 [file Image_1.pdf]

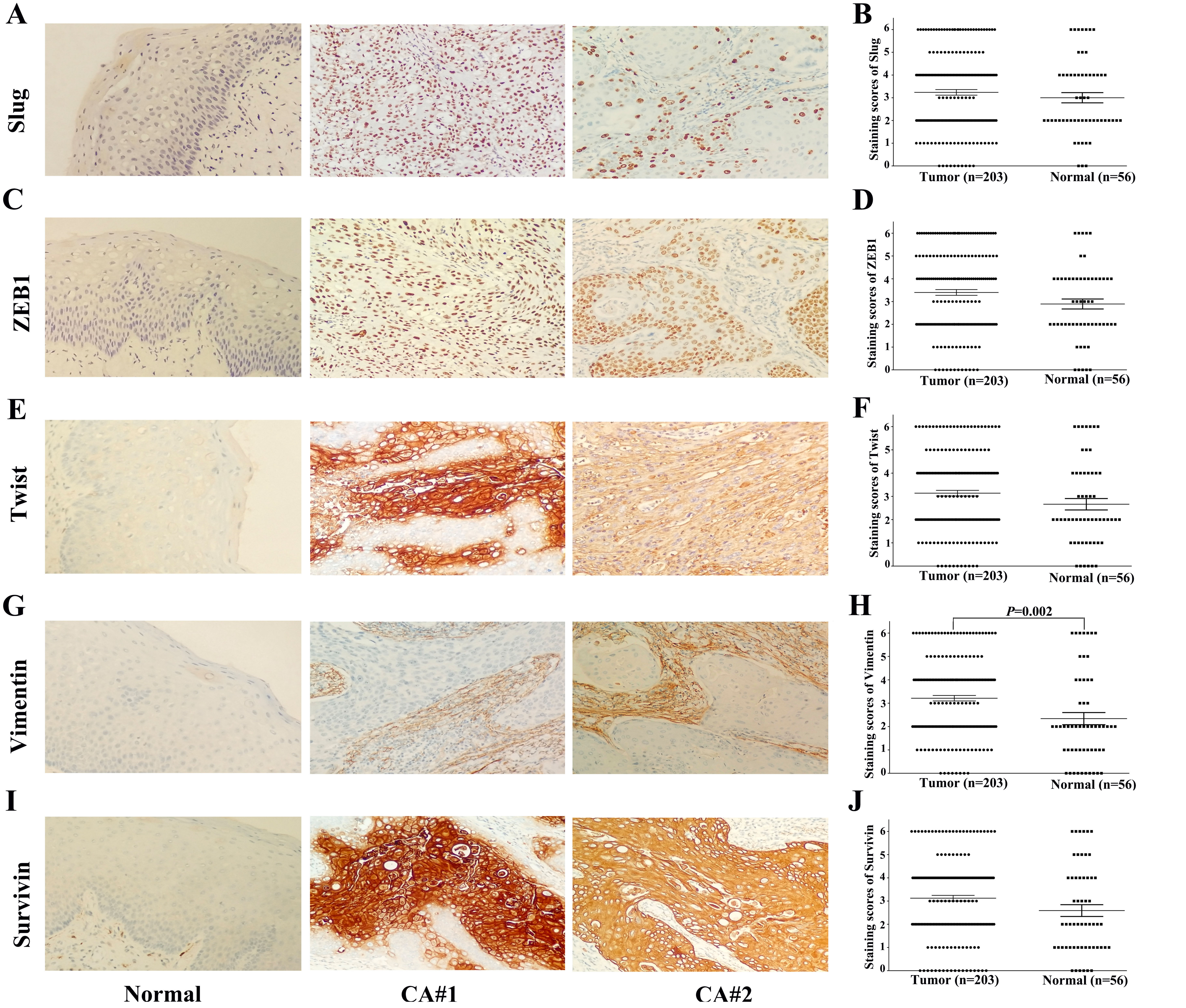

FIGURE S1. Slug, ZEB1, Twist, Vimentin, and Survival expression in cervical carcinoma tissues determined by immunohistochemical staining. (original magnification ×200). (A) Representative Slug expression in tumor and normal tissues, positive expression located in the nuclei. (B) Scatter dot plot showing the staining score (mean ± SEM) of Slug in tumor and normal tissues using the paired t-test. P = 0.35; (C) Representative ZEB1 expression in tumor and normal tissues, positive expression located in the nuclei. (D) Scatter dot plot showing the staining score (mean ± SEM) of ZEB1 in tumor and normal tissues using the paired t-test. P = 0.29; (E) Representative Twist expression in tumor and normal tissues, positive expression located in the cytoplasm. (F) Scatter dot plot showing the staining score (mean ± SEM) of Twist in tumor and normal tissues using the paired t-test. P = 0.39; P = 0.08; (G) Representative Vimentin expression in tumor and normal tissues, positive expression located in the cytoplasm. (H) Scatter dot plot showing the staining score (mean ± SEM) of Vimentin in tumor and normal tissues using the paired t-test. P = 0.002; (I) Representative Survivin expression in tumor and normal tissues, positive expression located in the cytoplasm. (J) Scatter dot plot showing the staining score (mean ± SEM) of Survivin in tumor and normal tissues using the paired t-test. P = 0.09.
